# Serum Metabolite Profile Associated with Sex-Dependent Visceral Adiposity Index and Low Bone Mineral Density in a Mexican Population

**DOI:** 10.3390/metabo11090604

**Published:** 2021-09-06

**Authors:** Berenice Palacios-González, Guadalupe León-Reyes, Berenice Rivera-Paredez, Isabel Ibarra-González, Marcela Vela-Amieva, Yvonne N. Flores, Samuel Canizales-Quinteros, Jorge Salmerón, Rafael Velázquez-Cruz

**Affiliations:** 1 Scientific Bonding Unit, Medicine Faculty UNAM-INMEGEN, Mexico City 14610, Mexico; bpalacios@inmegen.gob.mx; 2 Genomics of Bone Metabolism Laboratory, National Institute of Genomic Medicine (INMEGEN), Mexico City 14610, Mexico; greyes@inmegen.gob.mx; 3 Research Center in Policies, Population and Health, School of Medicine, National Autonomous University of Mexico (UNAM), Mexico City 04510, Mexico; bereriveraparedez7@gmail.com (B.R.-P.); jorge.salmec@gmail.com (J.S.); 4 Institute of Biomedical Research, IIB-UNAM, Mexico City 04510, Mexico; icig@servidor.unam.mx; 5 Laboratory of Inborn Errors of Metabolism, National Pediatrics Institute (INP), Mexico City 04530, Mexico; dravelaamieva@yahoo.com; 6 Epidemiological and Health Services Research Unit, Morelos Mexican Institute of Social Security, Cuernavaca 62000, Mexico; ynflores@ucla.edu; 7 Department of Health Policy and Management and UCLA-Kaiser Permanente Center for Health Equity, Fielding School of Public Health, University of California, Los Angeles (UCLA), Los Angeles, CA 90095, USA; 8 UCLA Center for Cancer Prevention and Control Research, Fielding School of Public Health and Jonsson Comprehensive Cancer Center, Los Angeles, CA 90095, USA; 9 Unit of Genomics of Population Applied to Health, Faculty of Chemistry, National Autonomous University of Mexico (UNAM)/National Institute of Genomic Medicine (INMEGEN), Mexico City 14610, Mexico; scanizales@inmegen.gob.mx

**Keywords:** branched-chain amino acids, acylcarnitines, sexual dimorphism, bone mass, adiposity

## Abstract

Recent evidence shows that obesity correlates negatively with bone mass. However, traditional anthropometric measures such as body mass index could not discriminate visceral adipose tissue from subcutaneous adipose tissue. The visceral adiposity index (VAI) is a reliable sex-specified indicator of visceral adipose distribution and function. Thus, we aimed to identify metabolomic profiles associated with VAI and low bone mineral density (BMD). A total of 602 individuals from the Health Workers Cohort Study were included. Forty serum metabolites were measured using the targeted metabolomics approach, and multivariate regression models were used to test associations of metabolomic profiles with anthropometric, clinical, and biochemical parameters. The analysis showed a serum amino acid signature composed of glycine, leucine, arginine, valine, and acylcarnitines associated with high VAI and low BMD. In addition, we found a sex-dependent VAI in pathways related to primary bile acid biosynthesis, branched-chain amino acids, and the biosynthesis of pantothenate and coenzyme A (CoA). In conclusion, a metabolic profile differs by VAI and BMD status, and these changes are gender-dependent.

## 1. Introduction

Obesity is related to metabolic disturbances such as type 2 diabetes (T2D), hypertension, insulin resistance, and osteoporosis [1,2,3]. According to the World Health Organization (WHO), more than 1.9 billion adults are overweight, of which more than 650 million are obese [4]. The latest National Health and Nutrition Survey 2018 in Mexico (ENSANUT 2018) reported that the percentage of adults with overweight and obesity was 75.2% [5], and osteopenia and osteoporosis in 2019 was 56% and 16%, respectively [6]. Osteoporosis is a common metabolic bone disorder characterized by low bone mineral density (BMD) and microstructural deterioration of bone tissues, which increases bone fragility and the risk of fractures [7]. BMD is the standard predictor for evaluating the bone quality in clinical diagnosis of osteoporosis and fracture risk, and serves as a surrogate marker for evaluating the effectiveness of treatment for osteoporosis [8].

Obesity and osteoporosis have strong genetic determinants. The heritability for BMD is estimated at 50–90% [9], and for body mass index (BMI) is at >40% [10]. In addition, they have specific pathogenesis and a shared biological basis [11,12]. Diverse studies have suggested that visceral adiposity is negatively associated with bone microarchitecture [13,14]. Some potential mechanisms that might explain this association include: (1) visceral adiposity generates an increase in the production of proinflammatory cytokines that could promote osteoclast differentiation [15], (2) the visceral adiposity is associated with a reduction of serum 25(OH)D levels which negatively impact in the BMD [16], and (3) systemic changes in lipid and polar metabolites could promote the production of cytokines and differentiation of osteoclast altering bone metabolism [17].

In most studies, obesity is ascertained, resorting to BMI. The BMI is a ratio between the weight to the squared height (kg/m^2^) of a subject, used to approximate body fat percentage. However, it is not ideal for measuring obesity because it cannot differentiate between visceral and subcutaneous fat, leading to considerable misclassification. Due to the complex metabolic role of the adipose tissue, it is necessary to classify obesity based on body fat composition and distribution [18].

The Visceral Adiposity Index (VAI) has recently been proven to indicate adipose distribution and function that indirectly expresses adverse effects of obesity [19]. The VAI is a mathematical model, gender-specific, based on anthropometric [(BMI and waist circumference (WC)] and functional parameters [triglycerides (TG) and HDL-cholesterol (HDL-c)]. Earlier reports have proposed that VAI is a good predictor for insulin resistance [20], T2D [21,22], cardiometabolic risk [23], and disturbances in glucose and lipid metabolism [24]. So far, there are no cut-off points to classify individuals with a low and high score of VAI in BMD; however, several studies have reported tertiles, quartiles, or quintiles [20,21,22,23]. Moreover, the VAI efficiently substitutes imaging modalities for assessing adipose tissue distribution, such as computed tomography and magnetic resonance imaging, which are usually inconvenient and expensive; and have radiation hazards [25]. Therefore, the VAI can be utilized as a reliable surrogate marker for evaluating obesity and the effects of obesity on BMD.

Recently, studies of obesity [26,27,28] and osteoporosis [29,30] with a metabolomic approach have pointed out the dynamic profile of metabolic changes associated with disease progression by quantifying metabolites in biological samples [31]. These changes are part of the subclinical stages of the disease and form a functional imprint of these individuals’ present and future responses. Interestingly, studies show sex-specific metabolic differences; for example, women incorporate free fatty acids (FFAs) into TG and have lower circulating acylcarnitines, whereas men oxidize FFAs [24]. Thus, sex differences affect physiology of several diseases and are organ and parameter specific, influencing the metabolism and homeostasis of amino acids, fatty acids, and sugars linked to the onset of diseases [32].

Hence, to date, no studies have investigated the possible modification of serum metabolites and their relationship between VAI and BMD status sex-dependent in the Mexican population. Our current study aimed to assess the relationship between VAI and BMD to examine possible modifications in the composition of serum metabolites in Mexican individuals.

## 2. Results

### 2.1. Population Demographic and Clinical Characteristics

This study included 602 individuals from the Health Workers Cohort Study (HWCS). The median age in the overall population was 60 years; though, women were older than men (*p* = 0.003). The median of WC, blood pressure, and BMD were higher in men than women (*p* < 0.05), additional features are presented in Table 1. In this study, women possess higher VAI than men; however, this difference was not statistically significant. Furthermore, we categorize the population by BMD status (Table S1) and age (Table S2). We observed that individuals with low-BMD were older than individuals with normal-BMD. In addition, they had a lower median of BMI, WC, body fat proportion, and BMD, as well as less prevalence of obesity (both categorized as total population or by sex) (*p* < 0.05). When we categorized the demographics by age groups, we found that the oldest individuals (≥70 years) had medians highest of WC, body fat proportion, glucose, HDL-c, and blood pressure (*p* < 0.05); as well as a higher prevalence of overweight, impaired glucose tolerance, T2D and lower values of BMD.

### 2.2. Serum Metabolite Profile According to Sex

**Figure 1:**
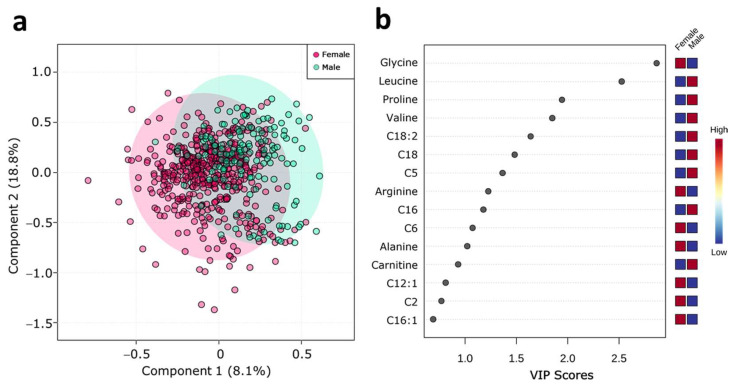
Serum metabolite profile according to sex. (**a**) PLS-DA plot shows separation between groups; female (red circles) and male (green circles). The explained variances are shown in brackets (accuracy: 0.80; R2: 0.24; Q2: 0.20: permutation *p*-value = 0.13); (**b**) VIP analysis represents the relative contribution of metabolites to the variance among groups. A high VIP score indicates a significant contribution of the metabolites to the group separation. Red and blue boxes indicate whether metabolite concentration is increased (red) or decreased (blue).

As mentioned above, sex-related considerations are increasingly being recognized in metabolic pathways and diseases. To understand sex contribution to metabolome, we performed a partial least-squares discriminant analysis (PLS-DA) and variable importance in projection (VIP) score. PLS-DA score plots separated subjects according to sex (Figure 1a). The VIP plot showed that glycine, leucine, proline, valine, octadecadienyl-carnitine, stearoyl-carnitine, isovaleryl-carnitine, arginine, palmitoyl-carnitine, hexanoyl-carnitine, alanine, and free carnitine (Figure 1b) are responsible for the separation between the groups.

### 2.3. Serum Metabolite Profile According to VAI

Although VAI, according to sex, did not have significant differences, it was borderline value (*p* = 0.06). Therefore, it was decided to categorize the VAI in tertiles (Table 2).

**Figure 2:**
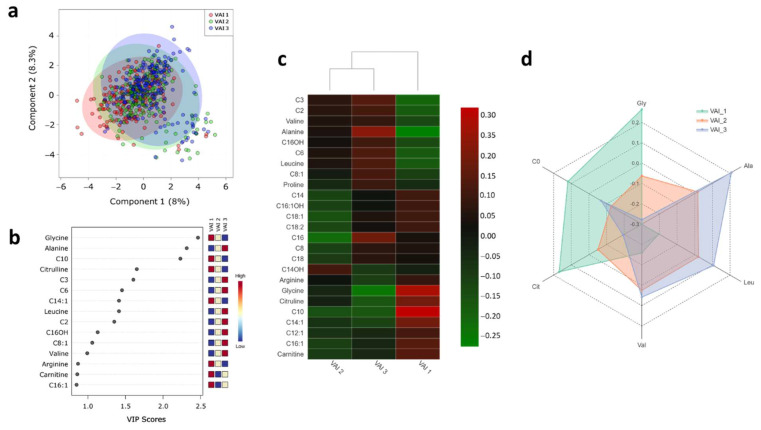
Serum metabolite profile according to visceral adiposity index. (**a**) PLS-DA plot shows separation between groups; VAI tertile 1 (red circles; reference group), VAI tertile 2 (green circles), and VAI tertile 3 (blue circles). The explained variances are shown in brackets (accuracy 0.43; R2 0.15; Q2 0.099: permutation *p*-value < 5.0 × 10^−4^); (**b**) VIP analysis represents the relative contribution of metabolites to the variance among groups. A high VIP score indicates a greater contribution of the metabolites to the group separation. Red and blue boxes indicate whether metabolite concentration is increased (red) or decreased (blue). (**c**) Hierarchical heatmap, red and green, indicate increase and decreased concentration, respectively. (**d**) Radar chart illustrating the abundance of amino acids and free carnitine among VAI tertiles: VAI tertile 1 (green; reference group), VAI tertile 2 (orange), and VAI tertile 3 (blue).

Regards serum metabolites, a total of 40 metabolites were quantified. The PLS-DA score plots revealed slight evidence of separation according to VAI tertiles (Q2 = 0.099: permutation test: *p*-value < 5.0 × 10^−4^) (Figure 2a). Despite this slight separation between the groups, the VIP plot showed those metabolites responsible for discrimination between the groups. Interestingly, glycine, alanine, decanoyl-carnitine, citrulline, propionyl-carnitine, hexanoyl-carnitine, tetradecenoyl-carnitine, leucine, acetyl-carnitine, and valine, showed VIP scores above 1.0 on VIP analysis, which made them potentially useful for discrimination (Figure 2b). Furthermore, unsupervised hierarchical clustering of abundance heatmap showed a separation between the VAI tertiles (Figure 2c). A distinctive pattern dependent on VAI tertile can be observed (Figure 2d). Briefly, the higher VAI, the higher the concentration of leucine, alanine, valine, and lower concentrations of glycine, free carnitine, and citrulline.

### 2.4. Metabolic Profile According to Sex-Dependent VAI

**Figure 3:**
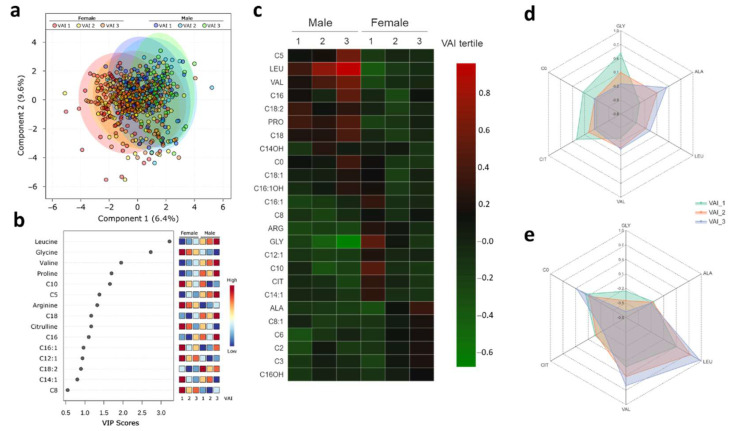
Metabolic profile according to sex-dependent visceral adiposity index. (**a**) PLS-DA plot shows discrimination between groups. Red circles represent females at first VAI tertile (VAI 1); yellow circles represent females at second VAI tertile (VAI 2); orange circles represent females at third VAI tertile (VAI 3); dark blue circles represent males at first VAI tertile (VAI 1); light blue circles represent males at second VAI tertile (VAI 2); green circles represent males at third VAI tertile (VAI 3).The explained variances are shown in brackets (accuracy 0.34; R2 0.19; Q2 0.15: permutation *p*-value < 5.0 × 10^−4^); (**b**) VIP analysis represents the relative contribution of metabolites to the variance among groups. A high VIP score indicates a greater contribution of the metabolites to the group separation. Red and blue boxes indicate whether metabolite concentration is increased (red) or decreased (blue). (**c**) Hierarchical heatmap, red and green, indicate increase and decreased concentration, respectively. (**d**) Radar chart illustrating the abundance of amino acids and free carnitine in females among VAI tertiles: VAI tertile 1 (green; reference group), VAI tertile2 (orange), and VAI tertile 3 (blue). (**e**) Radar chart illustrating the abundance of amino acids and free carnitine in males among VAI tertiles: VAI tertile 1 (green; reference group), VAI tertile 2 (orange), and VAI tertile 3 (blue).

Sex is also a determinant for the percentage and distribution of body fat. A PLS-DA was performed to evaluate a difference in VAI by sex (Figure 3a). After stratification according to sex, the metabolome was more distinct (Q2 0.15: permutation *p*-value < 5.0 × 10^−4^) compared to when only the VAI was evaluated (Q2 = 0.099). The VIP plot showed those metabolites responsible for the separation between the groups according to VAI and sex. Alanine, decanoyl-carnitine, glycine, hexanoyl-carnitine, propionyl-carnitine, acetyl-carnitine, citrulline, tetradecenoyl-carnitine, leucine, octenoyl-carnitine, hydroxyhexadecenoyl-carnitine, carnitine, and decanoyl-carnitine, showed VIP scores above 1.0 on VIP analysis (Figure 3b). Unsupervised hierarchical clustering of abundance heatmap showed a separation by sex and among the VAI groups (Figure 3c). Women with higher VAI had more leucine, alanine, valine and lower glycine, free carnitine, and citrulline concentrations than women with lower VAI (Figure 3d). Men with higher VAI had a greater abundance of leucine and valine and lower glycine and citrulline concentrations than men with lower VAI (Figure 3e).

### 2.5. Metabolite Set Enrichment Analysis

**Figure 4:**
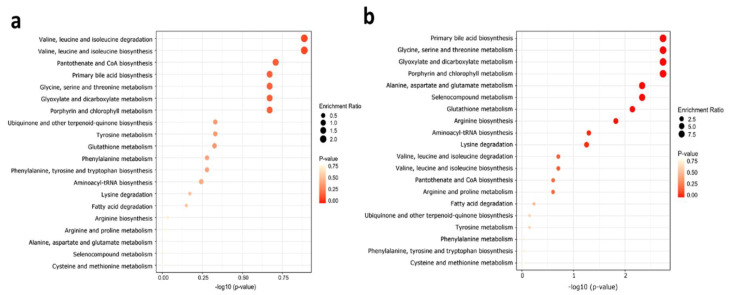
Dot plot of enriched metabolite pathway affected by VAI and sex. (**a**) Male VAI_1 (reference group), compared to VAI_2 and VAI_3 groups. (**b**) Female VAI_1 (reference group), compared to VAI_2 and VAI_3 groups. Colors indicate the *p*-values and the circles’ size based on the enrichment ratio.

To identify biologically meaningful patterns, we performed a metabolite set enrichment analysis (MSEA) in males and females with VAI_1 (reference group), compared to VAI_2 and VAI_3 groups, respectively (Figure 4a,b, respectively).

The metabolic pathways found include the metabolism of different amino acids, primary bile acid biosynthesis, pantothenate and coenzyme A (CoA) biosynthesis, glyoxylate and dicarboxylate metabolism, porphyrin, and chlorophyll metabolism, aminoacyl-tRNA biosynthesis, glutathione metabolism, and fatty acid degradation. The metabolites associated with these metabolic pathways are glycine, alanine, ornithine, arginine, citrulline, valine, leucine, carnitine, and palmitoyl-carnitine.

### 2.6. Metabolic Profile According to BMD Status

**Figure 5:**
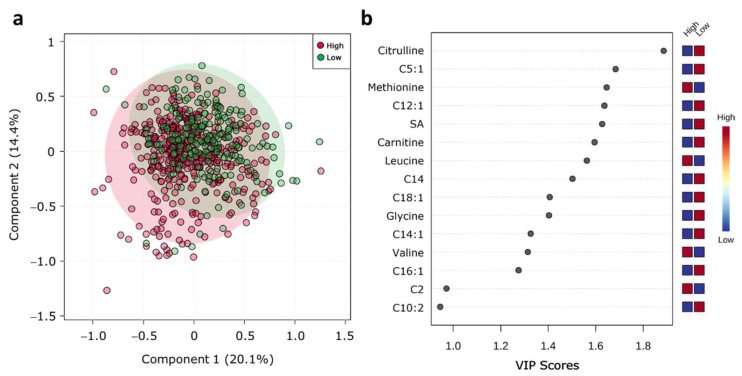
Serum metabolite profile according to BMD status. (**a**) PLS-DA plot shows separation between groups. Red circles represent individuals with high BMD; green circles represent individuals with low BMD. The explained variances are shown in brackets (accuracy 0.67; R2 0.11; Q2 0.069: permutation *p*-value < 5.0 × 10^−4^); (**b**) VIP analysis represents the relative contribution of metabolites to the variance among groups. A high VIP score indicates a greater contribution of the metabolites to the group separation. Red and blue boxes indicate whether metabolite concentration is increased (red) or decreased (blue).

Obesity is related to several metabolic diseases, such as osteoporosis, characterized by low-BMD. Thus, we categorize the population by BMD status (Table S1). To know if there was a difference in BMD status, a PLS-DA was performed. The PLS-DA score plot revealed a slight separation between high and low BMD clusters (Q2 = 0.069: permutation test: *p*-value < 5.0 × 10^−4^). Despite this slight separation between the groups, the VIP plot showed the citrulline, tiglyl-L-carnitine, methionine, dodecenoyl-carnitine, succinylacetone, free carnitine, leucine, and myristoyl-carnitine were associated with BMD status (Figure 5a,b).

### 2.7. Metabolic Profile According to Sex-Dependent VAI and BMD Status

**Figure 6:**
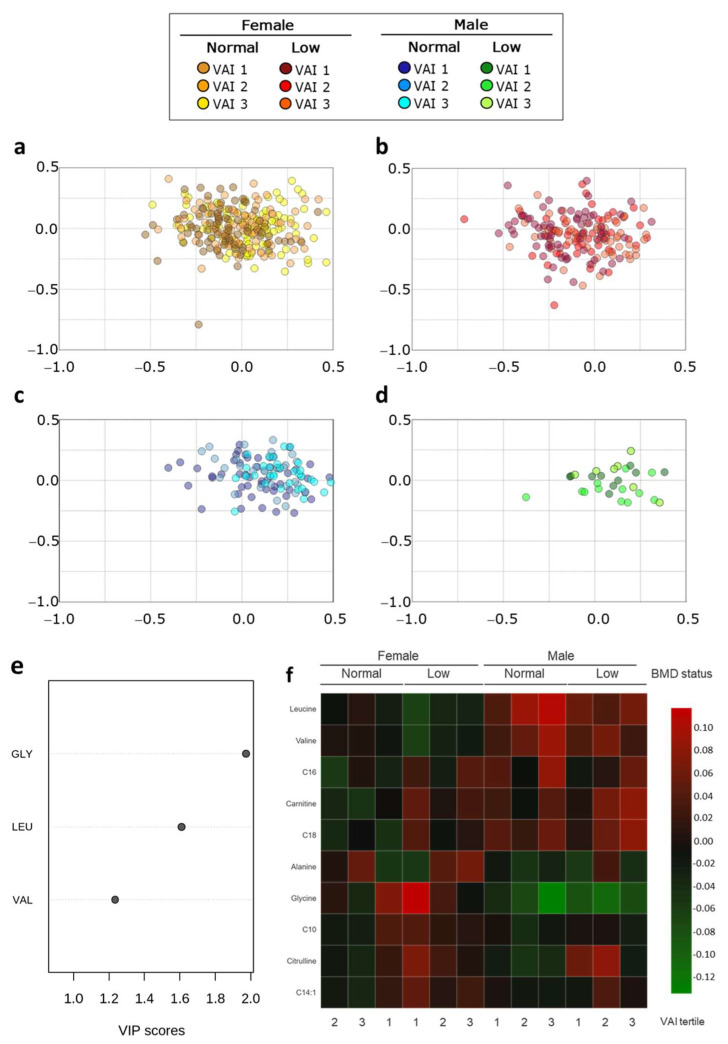
Metabolic profile according to sex-dependent VAI and BMD status. PLS-DA plot shows the separation between groups. The explained variances C1 = 26.3% and C2 = 18.8% (accuracy 0.21; R2 0.22; Q2 0.20: permutation *p*-value < 5.0 × 10^−4^); (**a**) female; normal BMD VAI1-VAI3, (**b**) female; low BMD VAI1-VAI3, (**c**) male; normal BMD VAI1-VAI3, (**d**) male; low BMD VAI1-VAI3. (**e**) VIP analysis represents the relative contribution of metabolites to the variance among the groups. A high VIP score indicates a greater contribution of the metabolites to the group separation. (**f**) Hierarchical heatmap, red and green, indicate increase and decreased concentration, respectively.

PLS-DA was performed to determine a metabolic profile related to sex-dependent VAI and BMD (Figure 6a–d). The VIP plot showed leucine, glycine, C10, valine, alanine, citrulline, free carnitine, C16, C14:1, and C18 responsible for separating BMD groups and VAI in a sex-dependent manner (Figure 6e). Unsupervised hierarchical clustering of abundance heatmap showed a separation by sex and among the VAI groups (Figure 6f). Briefly, leucine, valine, C16, free carnitine, and C18 tend to be higher in men regardless of BMD and VAI status. In addition, regardless of their VAI, women with low BMD have higher glycine, C10, citrulline, and C14:1 concentration. Interestingly, women with a VAI_1 and normal BMD present concentrations like women with low BMD. Interestingly, both men with low BMD and VAI_1/2 have similar concentrations of citrulline and C14:1.

## 3. Discussion

To the best of our knowledge, this is the first metabolomics study showing the serum profile of amino acids and free carnitines associated with the VAI and BMD status conducted in the Mexican population. We found that serum levels of branched-chain amino acids (BCAAs), glycine, citrulline, alanine, and free carnitine were significantly related to VAI. Our results are consistent with previous studies reporting impaired amino acids and carnitine metabolism in many metabolic diseases associated with obesity, such as hepatosteatosis, insulin resistance, and T2D [33,34,35,36,37,38,39]. Thus, high leucine, valine, and alanine concentrations were significantly associated with high VAI, whereas glycine levels were decreased. Studies in humans and rodents related to leucine and valine have indicated that an impaired adipose BCAA catabolic pathway contributes to the rise of plasma BCAAs and is associated with insulin resistance in obesity [40].

Otherwise, the literature indicates that elevated levels of BCAA interfere with the oxidation of fatty acids, leading to accumulation in the circulation of acylcarnitines. This assertion is in concordance with our results because we found that individuals with the highest level of VAI had increased levels of short- and medium-chain acylcarnitines and low levels of long-chain acylcarnitines. It has been proposed that the plasma concentrations of medium- and long-chain acylcarnitines can predict the intracellular energy metabolism pattern and be associated with the progression of several diseases, including insulin resistance, T2D, obesity, and cardiovascular disease [41,42,43,44].

In the present study, low glycine, arginine, and citrulline levels were associated with high VAI. Several studies have associated the non-essential amino acids (glycine and arginine), and citrulline, a metabolite of arginine, with an improved glycemic control [45,46,47], lower adiposity, and decreased risk factors for developing metabolic syndrome [48]. Interestingly, we found enrichment in glycine, serine, and threonine metabolism in our metabolic pathway analysis. Simmons et al. pointed out that a possible explanation for the low glycine concentrations in obese subjects could be the hepatic glycine cleavage system upregulation [49]. In addition, it has been described that arginine can activate AMPK and mTOR, resulting in greater insulin secretion and glucose uptake [50], thus being associated with better glycemic control.

On the other hand, citrulline is an amino acid synthesized mainly by enterocytes with antioxidant properties, participates in the urea cycle and the arginine and proline metabolism. Eshreif et al. indicated that this amino acid reduces muscle wasting and augmenting muscle performance. Moreover, it has been suggested that citrulline arises from elevations in mitochondrial function [51]. Ramírez-Zamora et al. highlighted an inverse correlation between plasma citrulline levels and glucose homeostasis in non-diabetic subjects [52]. In old rats, citrulline supplementation increased muscle mass, muscle fiber size, and the expression and activity of mitochondrial proteins [53]. Recently, Bouillanne et al. have also reported increased lean mass and decreased fat mass following citrulline supplementation [54]. The trophic effect of citrulline could be related to its ability to increase muscle apolipoprotein B editing complex 2 (APOBEC2) [53]. The preceding could indicate that those individuals in the highest tertile of the VAI would present lower muscle mass, which could have lower insulin sensitivity.

As mentioned above, sex is also a determinant for the distribution of body fat. In the present study, a differential metabolic pattern associated with VAI sex-dependent was observed. Women had lower amounts of BCAAs and higher concentrations of glycine and citrulline than men. These results are consistent with previous observations indicating that females have lower plasma levels of BCAAs than males [55,56]. Regulation of BCAA catabolism differs between sex; this could be due to hormonal influence [57]. It has been reported that elevated estrogen levels in women may partially contribute to the lower oxidation of amino acids than men [58]. Those findings highlight the need to consider sex differences when using these markers for risk assessment in metabolic disorders.

Another interesting finding from our study was the altered metabolite profile associated with BMD status, VAI, and sex. We identified six amino acids in serum, glycine, citrulline, leucine, arginine, valine, and several acylcarnitine significantly associated with high VAI and low BMD levels. Our findings are consistent with Su et al., which point out that higher serum valine level was associated with a lower risk of low BMD. A similar but weaker association was observed for leucine and isoleucine [59]. Previous reports indicate that amino acids play a crucial role in bone health and remodeling. Osteoporotic patients have lower circulating amino acids levels, which have been associated with low BMD. This association could be explained because amino acids modulate bone marrow stem cell (BMSC) function associated with signaling, proliferation, and differentiation in the bone marrow [17].

In the same way, carnitines play an essential role in bone metabolism. It has also been reported that 40% to 80% of energy demands in osteoclast and osteoclast are from fatty acid oxidation [60]. L-carnitine has been shown to stimulate human osteoblast functions and intracellular calcium signaling. Noteworthy, carnitines display a direct effect on human osteoblast by significantly increasing osteoblast activity and proliferation, as well as the expression of collagen type I, bone sialoproteins (BSPs), and osteopontin (OPN) [60].

Finally, the MESEA analysis showed that sex depending on VAI plays an essential role in enriched metabolic pathways, thus in men, the metabolism of BCAAs and the biosynthesis of pantothenate and CoA allow us to differentiate men in the lowest VAI of those in the top tertile. A urinary metabolomic study elucidated the perturbances of the high-fat diet on metabolomic profiling, consisted of higher concentrations of pantothenic acid and citrate and lower concentrations of proline and glycine [61]. The mechanism by which it is proposed that the concentrations of citrate and pantothenic acid are associated with weight gain is through the decrease in the ATP/AMP ratio, which promotes the inactivation of AMPK and the increase in lipogenesis [61].

Interestingly in the female low VAI group, top pathways were related to primary bile acid biosynthesis, glycine, serine, and threonine metabolism. Bile acids play a central role in the absorption of dietary lipid and have been recognized as essential modulators of glucose metabolism, insulin sensitivity, and energy expenditure [62]. Potential sex differences in bile acid homeostasis remain mostly unexplored. Recently, Baars et al. demonstrated a sex-specific regulation of bile acid homeostasis; thus, females showed higher serum concentrations of taurocholic acid (TCA), tauroalpha-muricholic acid (TAMCA), and taurobeta-muricholic acid (TBMCA) compared to males. The authors also suggest that these differences are partly microbiota-driven [63,64]. Several studies indicate apparent differences in lipid metabolism between sex, including cholesterol synthesis [65]. Our results point to alterations in lipid metabolism in women with a high VAI. The alterations are mainly in the metabolic pathways related to BCAAs and pantothenate and CoA biosynthesis in men.

The present study has several strengths. First, to our knowledge, this is the first such direct metabolomics study about VAI and BMD in the Mexican-Mestizo population. The population involved in our study is not only large (*n* = 602); however, it includes men and women with various statuses in terms of menopausal status and BMD loss progression, which raises the consistency of our results. Second, some of the metabolites (e.g., glycine, arginine, leucine, and valine) identified in this analysis have been previously associated with bone metabolism. This antecedent increases the confidence of our findings and may represent a valid and interesting metabolites-set associated with BMD. In addition, this motivates further studies to explore the identified metabolites in more detail. Third, we used the VAI, a recent extrapolated index that may be used as a surrogate marker of adipose tissue dysfunction, as a phenotype to maximize the identification of metabolites that could distinguish individuals at different risks for low BMD. There are, however, some limitations to this study. First, participants enrolled were recruited from the central region of Mexico (Cuernavaca, Morelos); therefore, additional studies are required before findings may be generalized to individuals in other areas of Mexico. Second, the sample size could limit the study to detect metabolites with minor effects on BMD. Third, we did not analyze the diet of individuals (one of the main determinants of human blood metabolites). We did not measure the main clinical biomarkers of bone metabolism, such as ALP, Trapcp5b, PINP, and CTX-I. Finally, the Neo-Base kit used does not determine isoleucine; therefore, the results on BCAAs may be taken with caution. Further studies are needed, including a broader panel of metabolites, to confirm our findings and clarify the potential molecular mechanisms of the detected associations between blood-identified metabolites, VAI, and BMD.

## 4. Materials and Methods

### 4.1. Study Population

This study is a cross-sectional analysis with data collected in the third wave (2016–2017) of the HWCS. The HWSC addresses a wide range of lifestyle and genetic factors to assess their association with several health outcomes. The details of this study cohort and methods have been previously described [66]. This cohort in the baseline measurement briefly recruited men and women ages 5 to 85 years from the Mexican Institute of Social Security (IMSS, by its acronym in Spanish) in Morelos, Mexico. This project was approved by the ethics committee of the IMSS. All participants signed informed consent. Exclusion criteria were individuals under 18 years of age or without a blood sample. A total of 602 individuals were included in this analysis.

### 4.2. BMD Measurements

Participants underwent dual-energy X-ray absorptiometry (DXA; Lunar DPX-GE, Lunar Radiation, software version 1.35, fast scan mode) to determine BMD at multiple sites (femoral neck, hip, and lumbar spine) in g/cm^2^ and their T-score [66]. The procedures were performed according to the manufacturer’s instructions. Standardized densitometry technicians ensured that the daily variation coefficient was within normal operational standards and the in vivo variation coefficient was lower than 1.5%. According to WHO criteria, low BMD was defined as a T-score less than −1 at the total hip [67].

### 4.3. Other Measurements

Demographic (age and sex), clinical (prior diagnosis of T2D), and lifestyle data (leisure-time physical activity) were obtained through self-administered questionnaires [66]. Height was measured without shoes on a standing stadiometer (SECA). Weight was measured using a calibrated electronic scale (BC-533; Tanita) with minimal clothing and bare feet. BMI was calculated as weight [kg/height (m)^2^]. Following exhalation, WC was determined with a tape measure to the nearest 0.1 cm at the top of the iliac crest.

Fasting plasma glucose, TG, and HDL-c were processed using a Selectra XL instrument (Randox), concordance with the International Federation of Clinical Chemistry and Laboratory Medicine [68].

VAI is a sex-specific index that was determined using the following equations [23]:

$$VAI_{men}:\left[\frac{WC_{cm}}{39.68+\left(1.88\times BMI\right)}\right]\times \frac{TG_{mmol/L}}{1.03}\times \frac{1.31}{HDL_{mmol/L}}$$

$$VAI_{women}:\left[\frac{WC_{cm}}{36.58+\left(1.89\times BMI\right)}\right]\times \frac{TG_{mmol/L}}{0.81}\times \frac{1.52}{HDL_{mmol/LH}}$$

All participants were divided into three groups based on *VAI* tertiles.

### 4.4. Metabolomics Analysis

Concentrations of serum acyl-carnitines, free carnitine, and amino acids were measured using the approach of targeted metabolomics by electrospray tandem mass spectrometry (Quattro Micro API tandem MS, Waters Inc., Milford, MA, USA). Metabolite levels in serum were analyzed using the commercial kit (NeoBase Non-derivatized MS/MS Kit, Perkin Elmer, Waltham, MA, USA), as previously described [69]. NeoBase MSMS kit is intended for the quantitative determination of Acylcarnitines: C0, C2, C3, C6DC, C4, C5, C5:1, C6, C8, C8:1, C16, C16:1, C16:1OH, C16OH, C10, C10:1, C10:2, C12, C12:1, C14, C14:1, C14:2, C14OH, C18, C18:1, C18:1OH, C18:2 and C18OH. Amino acids: Glycine, Alanine, Valine, Leucine, Methionine, Phenylalanine, Tyrosine, Ornithine, Citrulline, Arginine, and Proline. Ketone: Succinylacetone. Briefly, 20 µL of serum were poured onto filter paper cards (Whatman 903, Dassel, Germany) and dried at room temperature. The spot was cut into 2-mm circles and placed in a 96-well plate. The extraction solution was added to the plate and incubated for 30 min at 30 °C at 650× *g*. Finally, 10 µL of each sample were injected into the flow at 4-min intervals. The Micromass Quattro equipment (Waters Inc., Milford, MA, USA) was coupled with an ESI source in positive mode. Nitrogen gas was used for desolvation and nebulization, and argon was the collision gas.

### 4.5. Statistics

Descriptive analysis by sex and BMD status was performed using the Stata 14.0. Categorical data are presented as percentage and continuous data as median and 25–75th percentiles shown in parentheses. Proportion tests and Wilcoxon–Mann–Whitney tests were used to compare demographic and clinical characteristics by sex and BMD status, as appropriate. PLS-DA was used to visualize discrimination among samples. Permutation testing was carried out to minimize the possibility that the observed separation on PLS-DA was by chance. In addition to cross-validation, model validation was also performed by a 2000 times permutation test. A loading scatter plot was constructed to determine the variables discriminating between the groups. A VIP plot was performed for ranking the metabolites based on their importance in discriminating studies from both groups. VIP cutoff >1.0 was selected since the number of variables in this study was less than 100. We performed an MSEA to confirm biologically meaningful patterns between subjects. All statistical analyses were performed in Metabo Analyst 5.0 (McGill University, Toronto, ON, Canada). PLS-DA is a versatile algorithm used for predictive and descriptive modeling and the discriminative variable selection, which was performed to identify independent predictors that best correlated with VAI and bone status.

## 5. Conclusions

For the first time, this study has presented a cross-sectional assessment of a subset of metabolites (forty compounds) in serum obtained from Mexican men and women, using a metabolomic approach. Furthermore, we identified a metabolomic profile that varied among individuals with different VAI and BMD status, and ever these changes are gender-dependent. We identified leucine, glycine, C10, valine, alanine, citrulline, free carnitine, C16, C14:1, and C18 serum metabolites that distinguished the female and men groups, according to VAI and BMD in a Mexican population. This study adds evidence about the importance of amino acids and bile acids in obesity, providing new and additional clues to the pathogenic studies of gender and obesity.

## Figures and Tables

**Table 1:** Demographics of individuals belonging to the Health Workers Cohort Study.

	Total	Men	Women	*p*-Value
	*n* = 602	*n* = 145	*n* = 457	
Age (years) *	60 (50–68)	56 (46–65)	60 (52–68)	0.003
Age Categories, %				
<30 years	5.2	8.3	4.2	0.052
30–39 years	6.3	5.5	6.6	0.636
40–49 years	11.6	15.9	10.3	0.067
50–59 years	26.7	29.7	25.8	0.355
60–69 years	29.6	24.1	31.3	0.098
>70 years	20.6	16.6	21.9	0.169
BMI (kg/m^2^) *	26.9 (24.1–30.5)	26.5 (24.3–29.5)	27.1 (23.8–30.8)	0.439
Nutritional Status, %				
Overweight	39	41.4	38.3	0.505
Obesity	27.2	24.1	28.2	0.334
Waist circumference (cm) *	93 (86–100)	97 (91–105)	91 (84–99)	<0.001
Body fat proportion *	42.9 (37.0–47.9)	32.3 (28.6–36.3)	45.3 (40.5–49.8)	<0.001
Leisure time physical activity (min/day) *	12.9 (3.2–30)	12.9 (3.2–47.1)	12.8 (3.2–30.0)	0.06
Active (≥150/week), %	28.7	32.4	27.6	0.818
Missing, %	15.9	15.2	16	-
Glucose (mg/dL) *	99 (92–109)	101 (93–110)	98 (91–109)	0.162
Impaired Glucose tolerance (≥100–<126 mg/dL), %	32.1	35.9	30.9	0.261
Type 2 diabetes, %	18.3	20.7	17.5	0.385
Total cholesterol (mg/dL) *	197.5 (169–224)	196 (162–220)	198 (172–225)	0.096
Triglyceride (mg/dL) *	141 (105–197)	148 (106–207)	138 (105–194)	0.157
HDL-C (mg/dL) *	50.7 (42.3–59.8)	44.6 (38.7–52.2)	52.8 (45.2–61.9)	<0.001
LDL-C (mg/dL) *	113.1 (90.9–135.8)	113.2 (89.4–135.9)	113.1 (91.6–135.6)	0.785
Systolic blood pressure (mmHg) *	120 (109–134)	123 (114–137)	118 (107–133)	0.0004
Diastolic blood pressure (mmHg) *	75 (69–82)	79 (73–85)	74 (68–80)	<0.001
Femoral neck-BMD (g/cm^2^) *	0.91 (0.81–1.01)	0.99 (0.89–1.16)	0.88 (0.78–0.98)	<0.001
Lumbar spine-BMD (g/cm^2^) *	1.07 (0.95–1.18)	1.14 (1.06–1.27)	1.04 (0.93–1.15)	<0.001
Visceral Adiposity Index *	2.2 (1.5–3.3)	2.0 (1.4–3.2)	2.2 (1.5–3.4)	0.063

* Median (P25–P75). *p* value from the Wilcoxon–Mann–Whitney tests.

**Table 2:** Demographics classified by tertiles of the visceral adiposity index.

	VAI			
	Tertile 1	Tertile 2	Tertile 3	*p*-Value
	*n* = 201	*n* = 201	*n* = 200	
Sex, %				
Women	71.1	76.1	80.5	0.003
Age (years) *	58 (47–68)	61 (51–68)	60 (52–68)	0.043
Age Categories, %				
<30 years	7.5	3.5	4.5	0.206
30–39 years	10	6	3	0.005
40–49 years	11	11	13	0.538
50–59 years	26.4	26.9	27	0.892
60–69 years	23.4	33.8	31.5	0.069
>70 years	21.9	18.9	21	0.083
BMI (kg/m^2^) *	25.2 (23.0–28.1)	27.3 (24.5–30.8)	28.3 (25.8–31.8)	0.043
Nutritional Status, %				
Overweight	34.3	37.8	45	0.028
Obesity	18.4	28.9	34.5	0.0003
Waist circumference (cm) *	89 (82–96)	94 (87–100)	96 (88–104.5)	<0.001
Body fat proportion *	41.1 (33.3–46.1)	43.3 (36.3–49.1)	45.6 (38.9–49.0)	0.0001
Leisure time physical activity (min/day) *	12.9 (3.2–42.9)	7.7 (3.2–30.0)	12.9 (3.2–30.0)	0.145
Active (≥150/week), %	31.3	24.4	30.5	0.862
Missing, %	20.9	14.9	11.5	0.011
Glucose (mg/dL)	96 (88–102)	99 (91–108)	104 (95–121)	<0.001
Impaired glucose tolerance (≥100–<126 mg/dL), %	24.9	32.3	39	0.003
Type 2 diabetes, %	13.4	16.9	24.5	0.005
Total cholesterol (mg/dL) *	193 (163–214)	198 (167–225)	201 (177–236)	0.0008
Triglyceride (mg/dL) *	95 (72–113)	141 (121–164)	224 (186–283)	<0.001
HDL-C (mg/dL) *	60 (52–70)	51 (44–57)	43 (37–50)	<0.001
LDL-C (mg/dL) *	111 (90–132)	118 (94–141)	112 (87–133)	0.344
Systolic blood pressure (mmHg) *	116 (108–132)	120 (109–133)	122 (111–136)	0.013
Diastolic blood pressure (mmHg) *	74 (69–80)	74 (69–82)	77 (70–83)	0.014
Femoral neck-BMD (g/cm^2^) *	0.89 (0.80–1.02)	0.92 (0.81–1.01)	0.92 (0.81–1.01)	0.209
Lumbar spine-BMD (g/cm^2^) *	1.08 (0.97–1.19)	1.06 (0.95–1.17)	1.06 (0.95–1.16)	0.212

* Median (P25–P75). *p*-value for comparisons between tertile 1 and tertile 3. *p*-value from the Wilcoxon–Mann–Whitney tests.

## Data Availability

The data presented in this study are available in the article or Supplementary Material.

## Supplementary Materials

The following are available online at https://www.mdpi.com/article/10.3390/metabo11090604/s1, Table S1: Demographics categorized by sex and bone mineral density status of individuals belonging to the Health Workers Cohort Study, Table S2: Clinical and demographic data categorized by age groups, Table S3: Database from HWCS used in the study, Table S4: Description of analyzed metabolites.
