# Supplementary material for: Serum Metabolite Profile Associated with Sex-Dependent Visceral Adiposity Index and Low Bone Mineral Density in a Mexican Population
Source: Metabolites. 2021 Sep 6;11(9):604. doi: 10.3390/metabo11090604 (PMC8472083; doi:10.3390/metabo11090604)
Supplement: Supplementary file 1 [file metabolites-11-00604-s001.zip › Table S1.pdf]

Table S1. Demographics categorized by sex and bone mineral density status of individuals belonging to the Health Workers Cohort Study.

	Total			Men			Women		
	**Normal-BMD n=396**	**Low-BMD n=206**	***p*-Value**	**Normal-BMD n=115**	**Low-BMD n=30**	***p*-Value**	**Normal-BMD n=281**	**Low-BMD n=176**	***p*-Value**
Age(years)*	56(46-63)	67(59-73)	<0.001	55(44-62)	62(54-69)	0.006	57(48-63)	68(60-74)	<0.001
Age categories, %									
<30 years	7.1	1.5	0.003	10.4	0	0.065	5.7	1.7	0.037
30-39 years	8.3	2.4	0.005	7	0	0.136	8.9	2.8	0.01
40-49 years	14.7	5.8	0.001	16.5	13.3	0.669	13.9	4.6	0.002
50-59 years	32.1	16.5	<0.001	30.4	26.7	0.0693	32.7	14.8	<0.001
60-69 years	25.8	36.9	0.005	20.9	36.7	0.072	27.8	36.9	0.041
>70 years	12.1	36.9	<0.001	14.8	23.3	0.265	11	39.2	<0.001
BMI (kg/m^2)*	27.8(24.8-31.7)	25.2(23.0-27.8)	<0.001	27.1(24.5-30.4)	24.9(22.8-26.9)	0.0021	28.3(24.9-32.2)	25.6(23.0-28.0)	<0.001
Nutritional Status, %									
Overweight	38.9	39.3	0.924	41.7	40	0.866	37.7	39.2	0.748
Obesity	34.6	13.1	<0.001	28.7	6.7	0.012	37	14.2	<0.001
Waist circumference (cm) *	94(87-102)	89(83-97)	<0.001	98(92-106)	92(89-100)	0.018	93(85-101)	88(82-96)	0.0001
Body fat proportion*	43.2(36.3-49.2)	42.4(36.5-46.7)	0.178	33.1(29.3-36.9)	30.7(28.0-35.0)	0.128	46.4(41.6-50.6)	43.4(39.4-47.2)	0.0004
Leisure time physical activity (min/day) *	12.9(3.2-30.0)	12.9(3.2-30.0)	0.603	12.9(3.2-47.1)	16.0(4.8-36.4)	0.728	12.8(3.2-30.0)	12.8(2.3-30.0)	0.869
Active (≥150/week), %	27	32	0.198	31.3	36.7	0.574	25.3	31.3	0.163
Missing, %	17.9	12.6	-	17.4	6.7	-	17.4	13.6	-
Glucose (mg/dL)	100(91-112)	98(92-107)	0.12	101(92-110)	101(94-109)	0.817	100(91-112)	97(91-106)	0.12
Impaired Glucose tolerance (≥100-<126 mg/dL), %	33.3	29.6	0.356	36.5	33.3	0.745	32	29	0.499
Type 2 diabetes, %	20.2	14.6	0.092	20.9	20	-	19.9	13.6	-

Total cholesterol (mg/dL) *	197(165-222)	199.5(176-228)	0.134	193(160-219)	200(181-224)	0.296	198(170-225)	200(176-230)	0.373
Triglyceride (mg/dL) *	143(106-197)	206(105-194)	0.494	146(106-206)	155(106-212)	0.676	140(106-197)	136(105-189)	0.474
HDL-C(mg/dL) *	49.5(40.8-57.8)	53.1(45.8-65.1)	0.0001	43.8(38.1-53.0)	45.5(41.6-52.2)	0.293	51.8(43.4-60.0)	54.1(46.9-67.1)	0.003
LDL-C(mg/dL) *	112.0(89.3-135.9)	113.5(92.1-135.6)	0.378	110.9(84.9-135.6)	122.0(93.7-139.9)	0.232	112.7(91.6-136.3)	113.1(91.7-135.3)	0.715
Systolic blood pressure (mmHg) *	119(109-131)	120(108-138)	0.423	123(114-137)	122(112-140)	0.659	116(107-130)	120(108-137)	0.1
Diastolic blood pressure (mmHg) *	76(71-82.5)	74(67-80)	0.001	79(73-85)	77(72-87)	0.445	75(69-81)	74(67-79)	0.019
Femoral neck- BMD (g/cm^2)*	0.98(0.89-1.05)	0.77(0.71-0.82)	<0.001	1.03(0.96-1.17)	0.83(0.78-0.88)	<0.001	0.96(0.88-1.03)	0.76(0.71-0.81)	<0.001
Lumbar spine- BMD (g/cm^2) *	1.12(1.02-1.23)	0.95(0.85-1.04)	<0.001	1.18(1.10-1.30)	1.02(0.95-1.08)	<0.001	1.11(1.01-1.19)	0.92(0.84-1.03)	<0.001
Visceral Adiposity Index	2.2(1.5-3.5)	2.2(1.4-3.2)	0.293	2.0(1.4-3.3)	2.0(1.5-2.9)	0.781	2.3(1.6-3.6)	2.2(1.4-3.3)	0.113

* Median (P25-P75).
