# Supplementary material for: Serum Metabolite Profile Associated with Sex-Dependent Visceral Adiposity Index and Low Bone Mineral Density in a Mexican Population
Source: Metabolites. 2021 Sep 6;11(9):604. doi: 10.3390/metabo11090604 (PMC8472083; doi:10.3390/metabo11090604)
Supplement: Supplementary file 1 [file metabolites-11-00604-s001.zip › Table S2.pdf]

Table S2. Clinical and demographic data categorized by age groups.

	**<30 years n=31**	**30-<40 years n=38**	**40-<50 years n=70**	**50-<60 years n=161**	**60-<70 years n=178**	**≥70 years n=124**	***p*-value *(trend)***
BMI (kg/m^2) *	24.9(22.9-31.6)	25.5(22.5-29.8)	27.1(24.6-30.5)	26.9(23.8-31.1)	27.1(24.6-30.1)	27.2(24.4-29.7)	0.182
**Nutritional Status, %**							
Overweight	22.6	26.3	44.3	32.3	42.7	47.6	0.001
Obesity	25.8	23.7	25.7	32.9	25.8	24.2	0.204
Waist circumference (cm) *	88(77-103)	90(80-96)	93(85-99)	92(86-101)	93(85-99)	95(89-100)	0.006
Body fat proportion *	38.8(34.2-43.4)	42.2(36.9-48.1)	43.0(35.9-46.0)	41.5(34.1-47.6)	43.4(37.0-49.4)	44.3(38.6-46.7)	0.003
Leisure time physical activity (min/day) *	14.4(6.4-47.1)	11.2(1.4-25.7)	9.6(3.2-30.0)	12.9(3.2-30.0)	12.9(3.2-42.9)	8.6(3.2-30.0)	0.884
Active (≥150/week), %	29.0	18.4	24.3	26.1	34.3	29.8	0.278
Missing, %	22.6	21.1	18.6	17.4	11.8	14.5	-
Glucose (mg/dL)	90(85-96)	91(86-98)	95(90-104)	100(93-109)	100(95-114)	102(93-112)	<0.001
Impaired Glucose tolerance (≥100-<126 mg/dL), %	19.4	21.1	24.3	31.7	36.5	37.0	<0.001
Type 2 diabetes, %	-	2.6	12.9	21.7	19.1	25.0	<0.001
Total cholesterol (mg/dL) *	179.6(85.6-280.8)	170(110.5-247.7)	134.0(90.3-209.1)	133.7(81.7-204.3)	118.8(80.5-178.5)	120.9(73.6-195.0)	0.001
Triglyceride (mg/dL) *	104(73-190)	108(83-155)	141(106-198)	143(106-197)	150(114-203)	140(112-201)	0.002
HDL-C(mg/dL) *	46.7(39.1-54.4)	52.8(43.8-61.9)	47.2(38.7-56.1)	50.8(43.5-59.4)	51.6(43.2-59.8)	52.1(44.2-65.2)	0.012
LDL-C(mg/dL) *	94.4(80.0-110.9)	109.8(80.3-124.8)	113.3(86.1-132.4)	114.0(93.7-137.8)	118.3(95.5-139.0)	111.1(88.4-135.5)	0.015
Systolic blood pressure (mmHg) *	109(106-116)	108(102-115)	115(107-124)	117(108-127)	124(112-138)	132(119-147)	<0.001
Diastolic blood pressure (mmHg) *	71(65-80)	73(67-78)	77(71-84)	77(71-84)	76(69-82)	74(68-80)	0.937
Femoral neck- BMD (g/cm^2) *	1.12(1.02-1.25)	1.02(0.94-1.08)	1.01(0.92-1.11)	0.94(0.87-1.01)	0.87(0.78-0.95)	0.80(0.71-0.88)	<0.001
Lumbar spine- BMD (g/cm^2) *	1.22(1.12-1.25)	1.19(1.11-1.27)	1.16(1.07-1.27)	1.07(0.97-1.14)	1.01(0.90-1.12)	1.01(0.87-1.15)	<0.001
Visceral Adiposity Index	1.8(1.1-3.1)	1.6(1.1-2.5)	2.2(1.6-3.8)	2.2(1.5-3.5)	2.3(1.7-3.4)	2.3(1.5-3.6)	0.009

* Median (P25-P75).
